# Number of negative lymph nodes with a positive impact on survival of stage III colon cancer; a retrospective observation study for right side and left side colon

**DOI:** 10.1186/s12885-021-09154-z

**Published:** 2022-01-31

**Authors:** Yi-Hung Kuo, Jeng-Fu You, Hsin-Yuan Hung, Chih-Chien Chin, Jy-Ming Chiang, Chia-Hao Chang

**Affiliations:** 1 Division of Colon and Rectal Surgery, Department of Surgery, Chang Gung Medical Foundation, Chiayi Branch, No. 6, Sec. West, Chia–Pu Road, Putz City, Chiayi Hsien 613 Taiwan; 2 Graduate Institute of Clinical Medicine, Chang Gung University, Linkuo, Taiwan; 3 Division of Colon and Rectal Surgery, Department of Surgery, Chang Gung Medical Foundation, Linkuo, Taiwan; 4 Chang Gung University of Science and Technology, Chiayi, Taiwan

**Keywords:** Negative lymph node, Stage III colon cancer, Tumor location, Survival

## Abstract

**Background:**

The purpose was to examine the effect of negative lymph nodes (NLN) number on survival in stage III colon cancer. To reduce the interference of acute inflammation, we included patients with stage III colon cancer who had undergone elective surgery and excluded those who had tumor perforation, obstruction, ischemia, or massive tumor bleeding.

**Methods:**

This retrospective cohort study included 2244 patients with stage III colon cancer between 1995 and 2016 at a single center. The effect of NLN on 5-year relapse-free survival (RFS), 5-year overall survival (OS), and comparison of multivariate factors was assessed according to tumor locations.

**Results:**

The two optimal cutoff values of NLN for proximal and distal colon, namely 27 and 12, were determined by plotting the time-dependent receiver operating characteristic curve. Overall, 499 of 891 and 1020 of 1353 patients with right-side and left-side colon cancer, respectively, had high NLN. In right-side colon cancer, patients with high NLN (≥ 27) had superior OS (74.9% vs. 62.7%, *P* <  0.001) and RFS (75.0% vs. 61.9%, P <  0.001) than did those with low NLN. Moreover, in left-side colon cancer, patients with high NLN (≥12) experienced significantly superior OS (80.8% vs. 68.6%, *P* <  0.001) and RFS (77.3% vs. 66.2%, P <  0.001) than did those with low NLN. Among the different subgroups of stage III colon cancer, the high NLN group showed significantly superior RFS and OS in stage IIIB (RFS: 77.0% vs. 68.0%, *P* = 0.001; OS: 78.6% vs. 67.9%, *P* <  0.001) and IIIC (RFS: 58.2% vs. 44.1%, P = 0.001; OS: 65.7% vs. 51.1%, P <  0.001) colon cancer. However, in stage IIIA colon cancer, high NLN only showed survival benefit in OS (91.5% vs. 89.8%, *P* = 0.041). Multivariate analyses confirmed that high NLN, high carcinoembryonic antigen (≥ 5 ng/mL) level, and stage IIIC status are three independent prognostic factors in both the proximal and distal colon.

**Conclusions:**

NLN is a crucial prognostic factor for stage III colon cancer in various tumor locations or in the subgroups of stage III disease. In advanced stage III colon cancer, the importance of NLN and its role in anti-cancer immune response could be highlighted.

## A brief description

This study explored the relationship of negative lymph nodes (NLN) to the long-term outcome of stage III colon cancer. The impact of increasing number of NLN on survival prognosis was noted either in stage III colon cancer or in their subgroups.

## Background

Colorectal cancer (CRC) is the fourth most prevalent and the third most deadly cancer according to GLOBOCAN 2018 data [1]. After curative surgery, the tumor, node, and metastasis (TNM) stage, which is determined on the basis of the extent of tumor invasion and the status of lymph node metastasis and distant metastasis, is considered one of the most predictable prognostic factors [2]. In recent years, many studies have determined parameters associated with the lymph node status of CRC, including the number of lymph node harvest in stage II disease, metastatic lymph node ratio, and the number of negative lymph node (NLN) in stage III disease [3–9]. Some investigators believe that the more accurate stage of CRC could have resulted from increased lymph node examination, improved treatment quality, and advancement in surgical intervention or pathological evaluation. The number of NLN as a prognostic factor still is interesting when we consider the possible association between a patient’s immune response and their survival. Current evidence indicates that a suppressive cancer microenvironment formed due to interactions between tumor cells and other cells of the matrix, such as immune and nonimmune cells, is crucial for cancer development and progression [10–12]. Recently, a robust score quantification method for immune response was developed for clinical cancer classification. A low risk of cancer relapse was noted in patients with a high Immunoscore, which was quantified usingthe densities of CD3+ and cytotoxic CD8+ T cells in the tumor region and invasive margin. Patients’cancer recurrence at 5 years was 8, 19, and 32% in high, intermediate, and low Immunoscore groups [13].

Märkl et al. noted that lymph node size is a prognostic factor in node-negative colon cancer. The activation status of lymph nodes may be responsible for outcome differences associated with the number of lymph node yield in stage II colon cancer [14, 15]. Therefore, increased host’s immune response to tumors in CRC patients was hypothesized to be associated with a high number of NLN. In a large study performed using the Surveillance, Epidemiology, and End Result program (SEER) database, the association between the number of NLN and the prognosis of stage III colon cancer was analyzed [9]. In multivariate Cox regression, patients with the number of NLN ≥13 presented with significant survival advantage than did those with the number of NLN < 13. Up to date, few studies have analyzed the survival effect of the different numbers of NLN between right-side and left-side stage III colon cancer. Because a significant difference was noted in the number of lymph node yield in right-side and left-side colon cancer [16], the number of NLN for prognosis prediction could be different based on the location of stage III colon cancer. We conducted this retrospective study to assess the survival effect of the number of NLN on stage III colon cancer and its association with patients’ clinicopathological characteristics.

## Patients and methods

### Data sources

In total, 3034 patients with pathologic stage III colon cancer who underwent curative surgical resection at Chang Gung Memorial Hospital between 1995 and 2016 were initially enrolled in our analysis. Because an association existed between local or systemic inflammatory response syndrome and lymphadenopathy, only patients with stage III colon cancer without cancer obstruction, cancer perforation, ischemic colitis resulting from obstruction, or combinations of the aforementioned conditions were included in the present analysis. Finally, we excluded 790 patients and analyzed 2244 patients because of our study aim setting. Patients’ clinical and pathology data were collected prospectively and retrieved from the tumor registry of division of colon and rectal surgery. Follow-up data for survival were collected retrospectively according to medical record or interview. The last date of follow-up was February 28, 2020. In our data, no patient underwent preoperative radiotherapy or neoadjuvant chemotherapy. After selection, all matched cases had a mean follow-up of 64.5 months and a maximal follow-up of 154 months.

The clinicopathological data of each patient comprised their age; sex; preoperative laboratory data including Complete Blood Count/Differential Count carcinoembryonic antigen (CEA), and serum albumin; and cancer type, grade and TNM stage based on the Cancer Staging Manual, seventh edition, of American Joint Committee on Cancer. Tumor locations were divided into right side or proximal colon (proximal to the splenic flexure) and left side or distal colon (distal to the splenic flexure). All patients’ blood samples were obtained on admission for surgery. Appropriate approval for this observation study was obtained from the Institutional Review Board of the Chang Gung Medical Foundation (201701456B0).

### Survival follow-up and statistical analyses

In the study, categorical characteristics were compared using Pearson’s chi-squared test in right-side and left-side colon cancer. The survival difference was estimated using the Kaplan–Meier method, and a comparison was performed using the log-rank test. Overall survival (OS) was defined as the interval between the date of cancer diagnosis and the time of any-cause death. Relapse-free survival (RFS) was defined as the time from curative surgery to the disease relapse date. The 5-year OS and RFS were considered primary end points. The confounders were controlled for by using a Cox regression model in multivariate analysis. All statistical analyses were performed using SPSS version 17 (SPSS Inc., Chicago, IL, USA). All *P* values were two tailed and considered statistically significant if they were <  0.05.

**Fig. 1:**
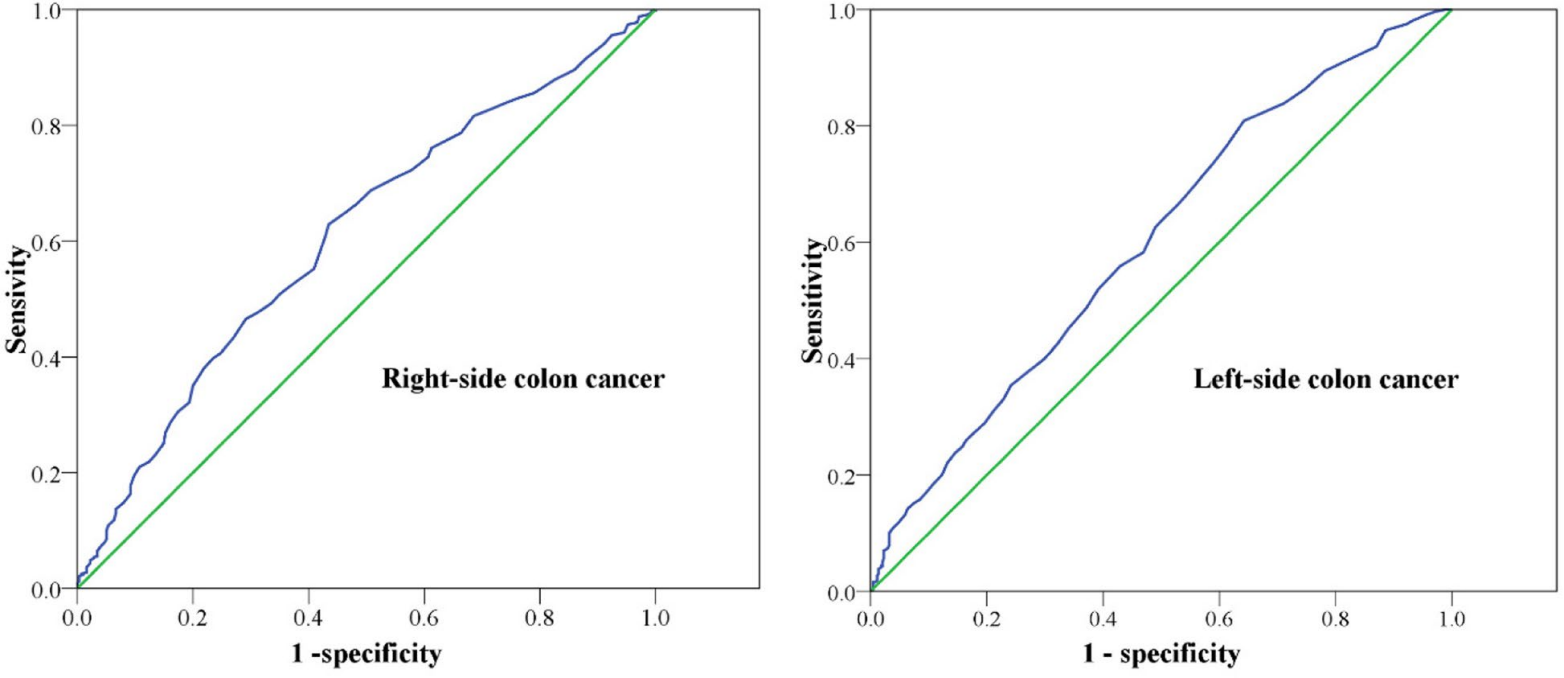
The time-dependent receiver operating characteristic **(**ROC) curve of the negative lymph node (NLN) counts for 5-year relapse-fee survival (RFS). Area under the ROC curve in right-side colon: 0.610 (95% confidence interval [CI]: 0.572–0.648, *P* < 0.001); area under the ROC curve for left-side colon: 0.602 (95% CI: 0.569–0.634, P < 0.001)

The association of NLN with oncology outcome in stage III colon cancer is unclear. Therefore, we analyzed the time-dependent receiver operating characteristic (ROC) curve to determine the optimal marginal value in our data. We focused on the ROC of NLN counts to determine patients’ RFS because colon cancer relapses are associated with patients’ overall survival [17]. The area under the ROC curve for the right-side colon was 0.610 (95% confidence interval [CI]: 0.572–0.648, *P* <  0.001), and that for the left-side colon was 0.602 (95% CI: 0.569–0.634, *P* <  0.001). According to the results of the ROC curve for 5-year RFS, the cutoff values of NLN in right-side and left-side colon cancer were 27 and 12, respectively (Fig. 1).

## Results

### Patient characteristics

The overall clinical characteristics of 2244 patients were as follows: patients’ median age at colon cancer diagnosis was 63 years (range, 22–99 years), and 50.4% of patients were men. The 891 right-side colon cancers tended to be more common in women (53.4%) than those in men (46.6%). In contrast to the gender distribution in right-side colon cancer, males (52.8%) were prominent than females (47.2%) in left-side colon cancer (*P* = 0.004, data not shown in Table). In pathology and tumor locations of all stage III colon cancers, right-side colon cancer accounted for 39.7% of enrolled patients. Among enrolled patients, 35.6 and 77.6% had abnormal CEA (≥5 ng/mL) and received adjuvant chemotherapy, respectively. The median numbers of examined lymph node (ELN), positive lymph node (PLN), and NLN were 26 (range, 2–154), 2 (range, 1–36), and 22 (range, 0–150), respectively, in the cohort of this study. The number of lymph node yield met the quality target(i.e. ≥12) in 94% of enrolled patients.

According to different tumor locations, the mean number and standard deviation of ELN were significantly higher in right-side colon cancer than in left-side colon cancer (35 ± 18 vs. 26 ± 15, *P* <  0.001). According to a previous study for reference data of the neutrophil-to-lymphocyte ratio (NLR) in Asia, an NLR of ≥2.87 was defined as abnormal [18]. The mean and standard deviation of NLR were 3.4 ± 3.3 and 2.9 ± 2.6 in stage III right-side and left-side colon cancer, respectively (*P* <  0.001).

To analyze the effect of the number of NLN on survival in stage III colon cancer, we defined optimal cutoff values for the number of NLN according to different tumor locations because significantly more ELN was noted in the right-side than in the left-side colon. Correlations between the NLN groups of different colon cancer locations and clinicopathological characteristics are summarized in Table 1. Overall, 499 of 891 and 1020 of 1353 patients with proximal and distal colon cancer, respectively, had high NLN. Patients with right-side as well as left-side colon cancer with a high number of NLN had a high proportion of pathological TNM − N1 stage. In right-side colon cancer, patients who received adjuvant chemotherapy after curative surgery (85.4% vs. 74.7, *P* <  0.001) and had adequate ELN (lymph node harvest ≥12, 100% vs. 89.6%, *P* <  0.001) were more in the high NLN group than in the low NLN group. Moreover, similar findings were presented in left-side colon cancer: adjuvant chemotherapy implementation (82.9% vs. 74.6%, *P* = 0.001) and adequate ELN proportion (number ≥ 12, 100.0% vs. 74.5%, *P* <  0.001) was significantly different between these two NLN groups.

**Table 1:** Categorical and continuous variables of patients with different numbers of NLN

	Right side colon, *n* = 891			Left side colon, *n* = 1353
Categorical variables	NLN ≥ 27 ***n*** = 499 (100%)	NLN < 27 ***n*** = 392(100%)	NLN ≥ 12 ***n*** = 1020 (100%)	NLN < 12 ***n*** = 333 (100%)
**Sex**, *P-value*	*0.069*		*0.254*	
Male	219 (43.9)	196 (50.0)	530 (52.0)	185 (55.6)
Female	280 (56.1)	196 (50.0)	490 (48.0)	148 (44.4)
**Age**, *P-value*	*0.001*		*0.002*	
Mean, SD (years)	62.2, ±13.5	65.2, ±13.6	61.5, ±13.4	64.1, ±12.8
**Age groups**, *P-value*	*0.004*		*0.010*	
< 50 year-old	86 (17.2)	53 (13.5)	185 (18.1)	44 (13.2)
50–65 year-old	188 (37.7)	115 (29.3)	408 (40.0)	116 (34.8)
65–80 year-old	177 (35.5)	171 (43.6)	339 (33.2)	140 (42.0)
≥80 year-old	48 (9.6)	53 (13.5)	88 (8.6)	33 (9.9)
**BMI**^a^, *P-value*	*0.184*		*0.226*	
Mean, SD	23.6, ±3.6	23.9,±3.9	24.2, ±3.7	24.5,±3.7
**BMI groups**^a^, *P-value*	*0.379*		*0.706*	
Underweight (BMI < 18.5)	31 (6.2)	24 (6.3)	45 (4.5)	11 (3.4)
Normal weight (18.5≦BMI < 24)	260 (52.3)	182 (47.8)	460 (45.5)	142 (44.0)
Overweight (24≦BMI < 27)	117 (23.5)	109 (28.6)	292 (28.9)	94 (29.1)
Obesity (BMI≧27)	89 (17.9)	66 (17.3)	214 (21.2)	76 (23.5)
**Pre-OP CEA**^a^, *P-value*	*0.566*		*0.715*	
Mean, SD (ng/mL)	11.2, ±24.5	12.3, ±31.5	8.7, ±20.4	9.1, ±19.0
**CEA groups**, *P-value*	*0.459*		*0.054*	
≥5 ng/mL	181 (37.0)	150 (39.5)	339 (33.5)	128 (39.4)
< 5 ng/mL	308 (63.0)	230 (60.5)	672 (66.5)	197 (60.6)
**Pre-OP NLR,** *P-value*	*0.219*		*0.007*	
Mean, SD	3.3, ±2.8	3.6, ±3.9	2.8, ±2.1	3.3, ±3.8
**NLR**,*P-value*	*0.650*		*0.060*	
≥2.87	200 (43.7)	143 (42.1)	284 (30.7)	100 (36.8)
< 2.87	258 (56.3)	197(57.9)	641 (69.3)	172 (63.2)
**Histology type**, *P-value*	*0.389*		*0.019*	
Adenocarcinoma	427 (85.6)	341 (87.0)	966 (94.7)	307 (92.2)
Signet ring cell	11 (2.2)	4 (1.0)	5 (0.5)	7 (2.1)
Mucinous	61 (12.2)	47 (12.0)	49 (4.8)	19 (5.7)
**Histology grade**, *P-value*	*0.028*		*0.771*	
Well differentiation	28 (5.6)	41 (10.5)	83 (8.1)	29 (8.7)
Moderate differentiation	379 (76.1)	284 (72.4)	872 (85.6)	279 (84.0)
Poorly differentiation	91 (18.3)	67 (17.1)	64 (6.3)	24 (7.2)
**T stage of TNM**, *P-value*	*< 0.001*		*< 0.001*	
T1	14 (2.8)	1 (0.3)	40 (3.9)	17 (5.1)
T2	17 (3.4)	17 (4.3)	80 (7.8)	36 (10.8)
T3	293 (58.7)	179 (45.7)	594 (58.2)	140 (42.0)
T4	175 (35.1)	195 (49.7)	306 (30.0)	140 (42.0)
**N stage of TNM**, *P-value*	*0.019*		*0.004*	
N1	349 (70.1)	245(62.5)	697(68.3)	199(59.8)
N2	150 (29.9)	147 (37.5)	323 (31.7)	134 (40.2)
**Stage III**, *P-value*	*0.045*		*< 0.001*	
IIIA	32 (5.8)	13 (3.9)	105 (10.3)	42 (12.6)
IIIB	409 (73.6)	225 (67.2)	720 (70.6)	195 (58.6)
IIIC	115 (20.7)	97 (29)	195 (19.1)	96 (28.8)
**Adjuvant C/T**, *P-value*	*< 0.001*		*0.001*	
Yes	446 (85.4)	242 (74.7)	807 (82.9)	247 (74.6)
No	76 (14.6)	82 (25.3)	166 (17.1)	84 (25.4)
**ELN,** *P-value*	*< 0.001*		*< 0.001*	
Mean, SD	46.5, ±15.2	20.6, ±6.5	30.2, ±13.9	11.4, ±4.3
**ELN group**, *P-value*	*< 0.001*		*< 0.001*	
**≥12**	556 (100.0)	300 (89.6)	1020 (100.0)	248 (74.5)
**PLN**, *P-value*	*0.002*		*< 0.001*	
Mean, SD	3.2, ±3.4	4.0, ±4.3	3.3, ±3.4	4.2, ±4.1
**NLN**, *P-value*	*< 0.001*		*< 0.001*	
Mean, SD	43.3, ±14.8	16.6, ±6.0	26.9, ±13.6	7.2, ±2.9

*ELN* examined lymph node number, *NLN* negative lymph node number, *PLN* positive lymph node, *BMI* body mass index, *TNM* tumor/node/metastasis classification, *N1 of TNM* metastasis in 1–3 regional lymph nodes, *N2 of TNM* metastasis in ≥4 regional lymph nodes, *Adjuvant C/T* adjuvant chemotherapy, *SD* standard deviation, *CEA* carcinoembryonic antigen, *NLR* neutrophil to lymphocyte ratio

^a^:missing data; 39 patients in CEA data and 13 patients in BMI data

### Survival analyses

**Fig. 2:**
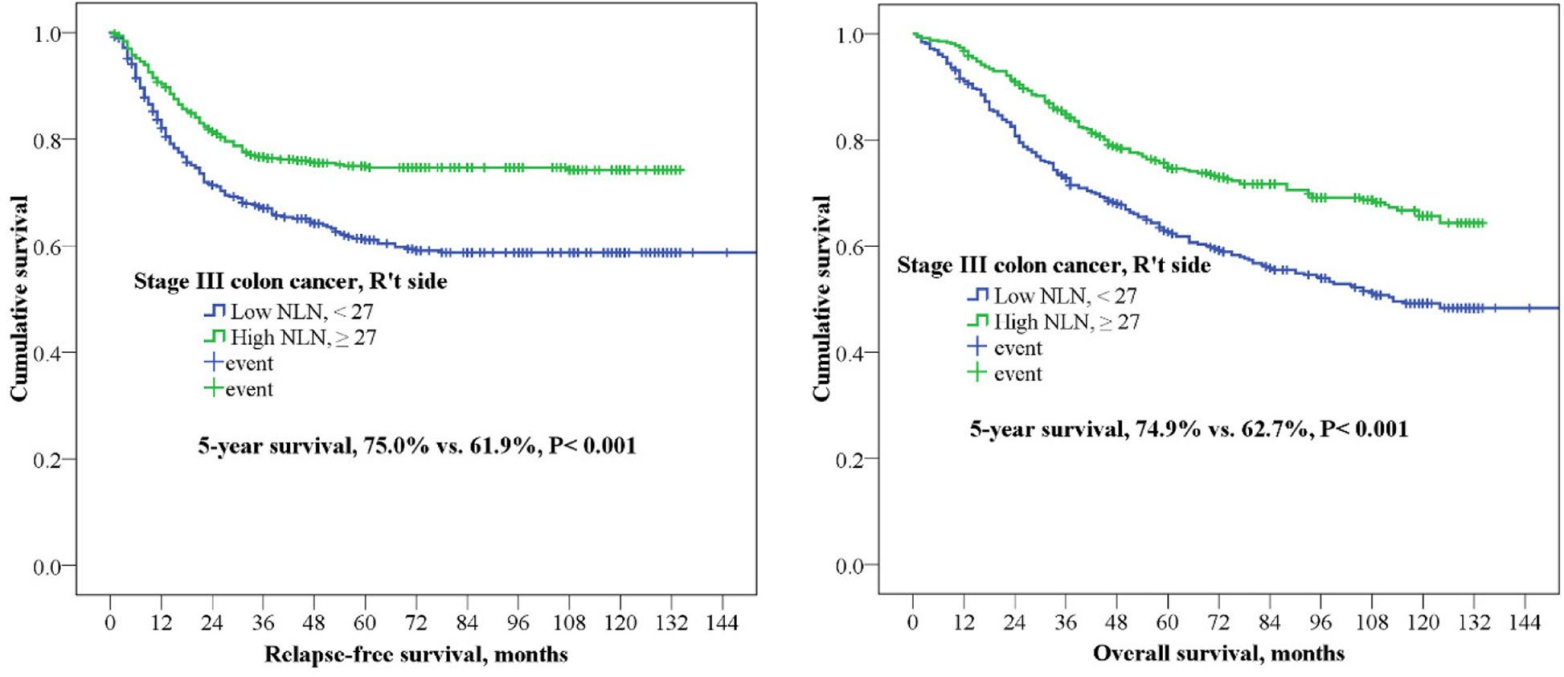
5-year relapse-fee survival (RFS) and 5-year overall survival (OS) for stage III colon cancer in right-side colon. Kaplan-Meier curve was according to number of nonmetastatic lymph nodes (NLN ≥27 vs. < 27)

**Fig. 3:**
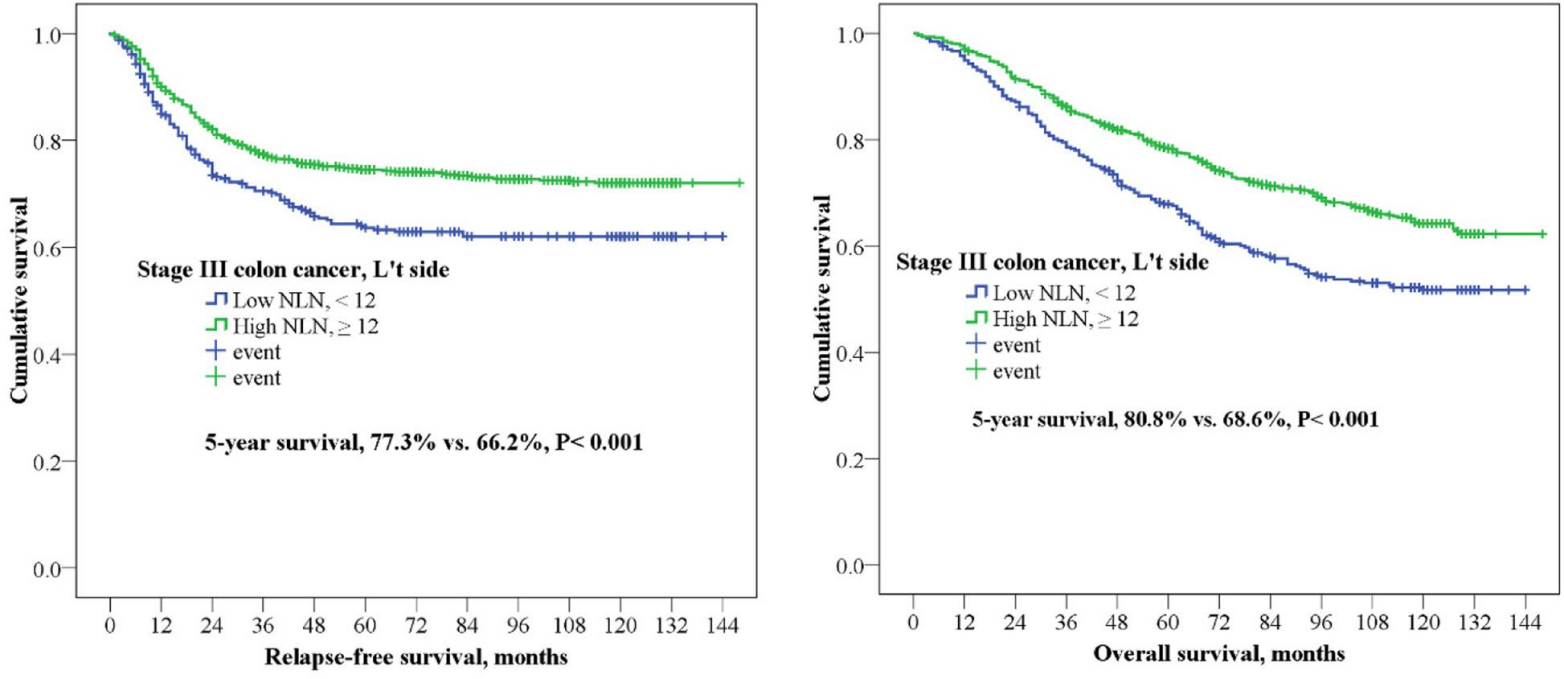
5-year relapse-fee survival (RFS) and 5-year overall survival (OS) for stage III colon cancer in leftt-side colon. Kaplan-Meier curve was according to number of nonmetastatic lymph nodes (NLN ≥12 vs. < 12)

In this study, the 5-year RFS and OS of right-side colon cancer were 62.3 and 69.4% and those of left-side colon cancer were 65.4 and 75.7%, respectively. In stage III colon cancer with high and low NLN, patients with a high number of NLN (≥27) in right-side colon cancer experienced a significantly better 5-year OS (74.9% vs. 62.7%, *P* <  0.001) and 5-year RFS (75.0% vs. 61.9%, *P* <  0.001) than did those with a low number of NLN (Fig. 2). Furthermore, similar results were noted in left-side colon cancer; that is, patients with a high number of NLN (≥12) experienced a significantly superior 5-year OS (80.8% vs. 68.6%, *P* <  0.001) and 5-year RFS (77.3% vs. 66.2%, *P* <  0.001) than did those with a low number of NLN (Fig. 3).

In a multivariate analysis of Cox regression for 5-year RFS and 5-year OS (Tables 2 and 3), the effect of a high number of NLN (≥27) in right-side colon cancer was independent of age, sex, CEA level, NLR, subgroups of stage III colon cancer, histology type and grade, and implementation of adjuvant chemotherapy after curative resection (RFS, hazard ratio [HR]: 0.613, 95% confidence interval [CI]: 0.463–0.812, *P* = 0.001; OS, HR: 0.626, 95% CI: 0.487–0.804, *P* <  0.001). Furthermore, a high number of NLN (≥12) in left-side colon cancer presented as an independent prognostic factor for RFS (HR: 0.705, 95% CI: 0.549–0.906, *P* = 0.006) and OS (HR: 0.655, 95% CI: 0.522–0.823, *P* <  0.001). A preoperative CEA level ≥ 5 ng/mL, a high number of NLN (≥27 in the right-side colon and ≥ 12 in the left-side colon), and TNM stage IIIC disease were three independent prognostic factors for RFS and OS in right-side and left-side stage III colon cancer.

**Table 2:** Multivariate analysis of Cox regression for 5-year RFS and OS in right-side colon cancer

Variables	RFS		OS	
	HR (95% CI)	P-value	HR (95% CI)	P-value
**Sex**				
Female	1		1	
Male	1.181 (0.898–1.554)	0.233	0.963 (0.753–1.231)	0.763
**Age**				
< 50 year-old	1		1	
50–65 year-old	1.434 (0.925–2.223)	0.107	1.144 (0.749–1.745)	0.534
65–80 year-old	1.114 (0.718–1.729)	0.630	1.361 (0.910–2.034)	0.134
≥80 year-old	1.249 (0.704–2.216)	0.446	2.951 (1.856–4.692)	< 0.001
**CEA**, pre-operation				
≥5 ng/mL	1		1	
< 5 ng/mL	0.611 (0.463–0.806)	< 0.001	0.717 (0.560–0.918)	0.008
**NLR**, pre-operation				
≥ 2.87	1		1	
< 2.87	1.054 (0.796–1.397)	0.713	0.864 (0.664–1.124)	0.277
**Histology type**				
Adenocarcinoma	1		1	
Signet ring cell	1.509 (0.528–4.313)	0.443	2.691 (1.128–6.418)	0.026
Mucinous	0.927 (0.582–1.477)	0.751	1.038(0.693–1.554)	0.856
**Histology grade**				
Poorly	1		1	
Moderate	1.231 (0.823–1.842)	0.311	1.262 (0.884–1.802)	0.200
Well	0.792 (0.396–1.583)	0.509	0.719 (0.381–1.356)	0.308
**NLN**				
< 27	1		1	
≥ 27	0.613 (0.463–0.812)	0.001	0.626 (0.487–0.804)	< 0.001
**Stage III**				
IIIA	1		1	
IIIB	8.661 (1.207–62.167)	0.032	1.990 (0.809–4.894)	0.134
IIIC	19.874 (2.746–143.811)	0.003	4.030 (1.612–10.076)	0.003
**Adjuvant C/T**				
No	1		1	
Yes	0.702 (0.484–1.018)	0.062	0.517 (0.387–0.690)	< 0.001

*RFS* relapse –free survival, *OS* overall survival, *CEA* carcinoembryonic antigen, *NLR* neutrophil to lymphocyte ratio, *NLN* negative lymph node number, *Adjuvant C/T* adjuvant chemotherapy

**Table 3:** Multivariate analysis of Cox regression for 5-year RFS and OS in left-side colon cancer

Variables	RFS		OS	
	HR (95% CI)	P-value	HR (95% CI)	P-value
**Sex**				
Female	1		1	
Male	1.265 (1.003–1.594)	0.047	1.352(1.085–1.683)	0.007
**Age**				
< 50 year-old	1		1	
50–65 year-old	0.865 (0.625–1.196)	0.380	0.881(0.607–1.278)	0.504
65–80 year-old	0.825 (0.589–1.156)	0.264	1.649(1.162–2.341)	0.005
≥80 year-old	0.809 (0.482–1.359)	0.424	2.191(1.424–3.370)	< 0.001
**CEA**, pre-operation				
≥5 ng/mL	1		1	
< 5 ng/mL	0.504 (0.400–0.634)	< 0.001	0.502(0.404–0.624)	< 0.001
**NLR**, pre-operation				
≥ 2.87	1		1	
< 2.87	0.906 (0.710–1.156)	0.425	0.697(0.558–0.870)	0.001
**Histology type**				
Adenocarcinoma	1		1	
Signet ring cell	0.918 (0.316–2.668)	0.876	1.213(0.429–3.426)	0.716
Mucinous	0.965 (0.575–1.620)	0.894	0.918(0.554–1.522)	0.740
**Histology grade**				
Poorly	1		1	
Moderate	0.658(0.422–1.026)	0.065	0.625(0.397–0.986)	0.044
Well	0.539(0.280–1.035)	0.063	0.647(0.354–1.183)	0.157
**NLN**				
< 12	1		1	
≥ 12	0.705 (0.549–0.906)	0.006	0.655(0.522–0.823)	< 0.001
**Stage III**				
IIIA	1		1	
IIIB	2.494 (1.339–4.642)	0.004	1.679 (1.049–2.686)	0.031
IIIC	4.414(2.318–8.408)	< 0.001	2.482 (1.495–4.122)	< 0.001
**Adjuvant C/T**				
No	1		1	
Yes	1.054 (0.750–1.482)	0.760	0.524(0.404–0.681)	< 0.001

*RFS* relapse –free survival, *OS* overall survival, *CEA* carcinoembryonic antigen, *NLR* neutrophil to lymphocyte ratio, *NLN* negative lymph node number, *Adjuvant C/T* adjuvant chemotherapy

**Fig. 4:**
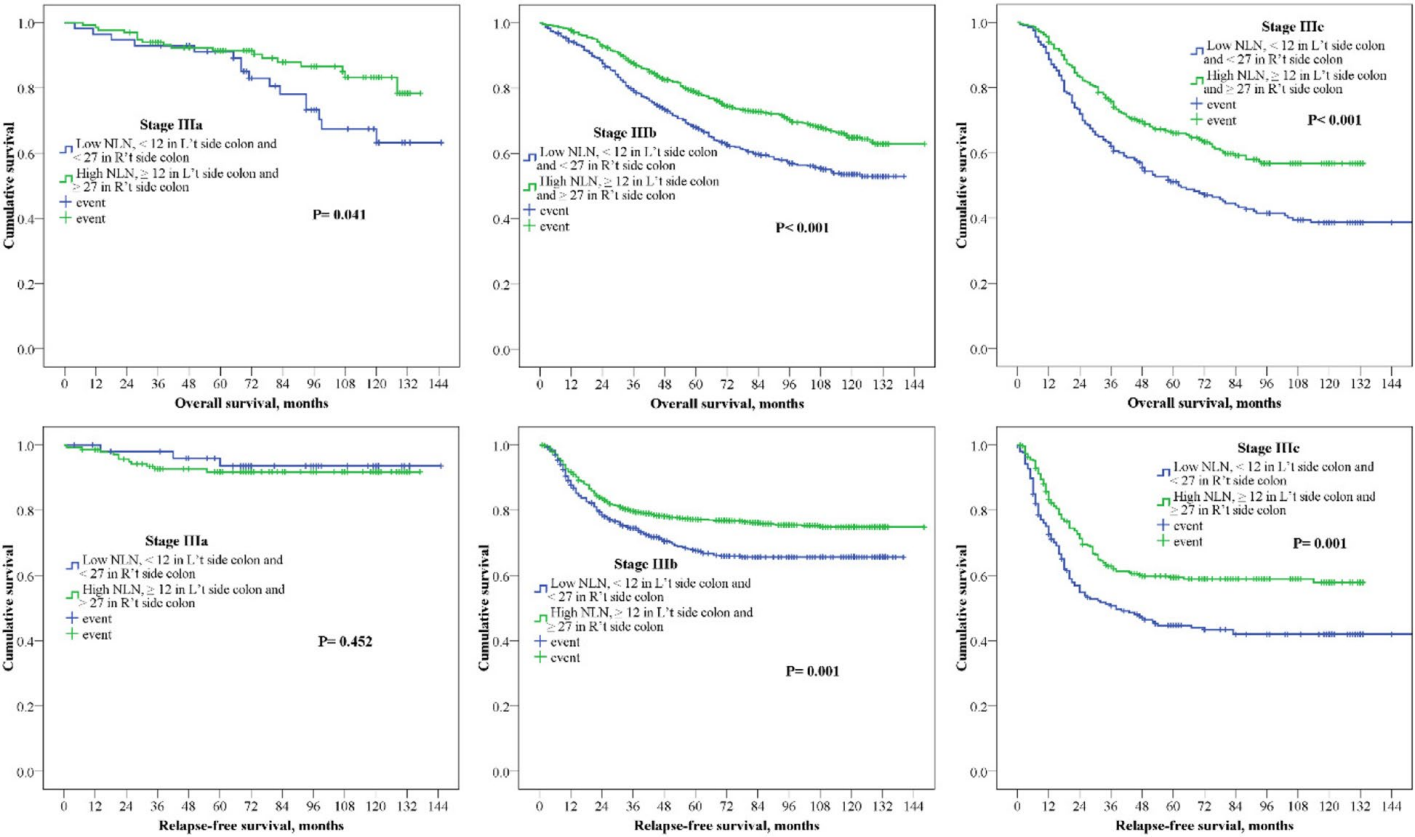
5-year relapse-fee survival (RFS) and 5-year overall survival (OS) for subgroup of stage III colon cancer, including stage IIIa, IIIb and IIIc. Kaplan-Meier curve was according to high NLN (NLN ≥12 in left-side colon or NLN ≥27 in right-side colon)

We further analyzed the effect of a high number of NLN on different subgroups of the whole stage III colon cancer, including stage IIIA, IIIB, and IIIC. Patients with a high number of NLN had significantly superior 5-year RFS and OS in stage IIIB (RFS: 77.0% vs. 68.0%, *P* = 0.001; OS: 78.6% vs. 67.9%, *P* <  0.001) and IIIC (RFS: 58.2% vs. 44.1%, *P* = 0.001; OS: 65.7% vs. 51.1%, *P* <  0.001) colon cancer (Fig. 4). In stage IIIA colon cancer, a high number of NLN showed survival benefit in only 5-year OS (91.5 vs. 89.8%, *P* = 0.041).

## Discussions

In the present study, an increased number of NLN (≥27 and ≥ 12 for right-side and left-side colon cancer, respectively) was significantly associated with increased 5-year RFS and OS of stage III colon cancer. Subgroup analysis results for stage IIIA, IIIB, and IIIC colon cancer showed that a high number of NLN was not significantly prognostic in only 5-year RFS of stage IIIA colon cancer. In stage IIIB and IIIC colon cancer, patients with a high number of NLN had significantly superior 5-year RFS than did those with a low number of NLN. The similar results were also reported by Paul et al. and Quan et al. who have recommended that the role of NLN in survival prognosis was prominent in right-side colon, stage IIIB and IIIC disease [9, 19]. These findings may highlight the influence of host immune response at different tumor invasion levels. The effect of lymph nodes dissection on prognosis has been acknowledged in the past decade. Age, tumor size, and advanced T stage are independent factors affecting the number of ELNs in colon cancer [20]. Age < 65 years and deep tumor invasion may result in strong antigenic immune activity and a strong inflammatory response. Furthermore, in our data, a high number of NLN was significantly associated with age < 65 years and T3/T4 invasion level. Tekkis et al. suggested a positive association between increased ELN and patients’ immunologic reactions depending on their age [21]. In the preoperative clinical stage and postoperative pathological stage, the degree of difficulty in evaluating or harvesting lymph nodes is influenced by lymph node size. Therefore, reactive lymph nodes probably increase the number of ELN in standard curative surgery.

In stage III colon cancer, the NLN has been found to be a prognostic factor, like the metastatic lymph node ratio [7, 9, 19]. Moreover, this independent effect of NLN was even reported in overall survival of stage IV colon cancer [22]. In previous studies on colorectal cancer, only approximately 26 of 100 lymph nodes greater than 10 mm had metastatic cancer [14, 23], meaning that most large lymph nodes were not associated with cancer. Therefore, lymph node size and number may be crucial in anticancer immune response. Patterns of NLN for colorectal cancer, which include follicular, parafollicular, and sinus histiocytosis, were regarded to be predictors of host immunity and associated with an improved prognosis [24]. Large tumor size and deep invasion are thought to induce an antigenic effect; consequently, lymphatic hyperplasia may be commonly found in regional lymph nodes. A high lymphatic reaction may increase the likelihood of lymph node enlargement. If many reactive lymph nodes are in a patient’s surgical specimen, a higher number of NLN can be detached early or easily. Additionally, lymphocyte infiltration into the tumor margin and central part was shown to be high in patients with a high NLN. Crohn-like reaction, which was the discrete aggregation of lymphoid white blood cells, some with germinal centers and surrounding fibrosis, was commonly found around some colorectal adenocarcinomas in the absence of a clinical or pathological diagnosis of Crohn disease [22]. This mechanism can further explain the different effects of high NLN on different cancer stages of our present study. In more advanced stage III colon cancer, the importance of anticancer immune response could be highlighted more significantly. That was probably why the positive effect of increased NLN was more significant on 5-year RFS and OS in stage IIIB/IIIC disease than it was on survival in stage IIIA disease.

In the multivariate Cox proportional hazard model of the present study, the high number of NLN in left-side and right-side colon cancer was an independent prognostic factor for RFS and OS. The cancer-relapse risk decreased more with a high number of NLN in right-side colon cancer than in left-side colon cancer (38.7% vs. 29.5%). Generally, the prognosis of left-side colon cancer was excellent compared with right-side colon cancer, and this advantage might weaken the statistic power in NLN analysis. According to immune response theory, greater number of NLN indicated better host immune defense. In the present study, up to 95 and 89.1% right-side and left-side colon cancer, respectively, were stage IIIB/IIIC. Therefore, the prognostic value of high NLN probably was enhanced and highlighted in right-side stage III colon cancer. In the present study, we examined only stage III colon cancer without any emergent conditions, including perforation, obstruction, ischemia, and massive tumor bleeding to minimize the effect of acute inflammation. We considered that the status of NLN of stage III colon cancer could reflect the real prognostic role for RFS and OS. Therefore, both 5-year RFS and OS were higher in the present study than in other reports, and differences in inclusion and exclusion criteria might have influenced this difference [19, 25, 26]. Besides, the classification of NLN is usually debatable. Unlike the CEA level or TNM stage system, the cutoff value of NLN still lacks a uniform standard. Moreover, exploring reference data, such as a study for NLR from a big healthy population, is challenging [18]. The ROC analysis is usually used to find the most discriminative cutoff value in such studies, and different threshold values were noted in previous published data, including 9 [19] and 13 [9, 22]. Because the distribution of lymph nodes in the mesocolon varies according to colon cancer location, the number of lymph node harvest in colon cancer surgery is usually higher in the right-side than in the left-side colon [27]. The single-threshold value of NLN possibly did not present the potential difference in lymph node harvest, which could be associated with different colonic locations (e.g., proximal colon cancer vs. distal colon cancer), different curative procedures (e.g., right hemicolectomy vs. anterior resection), and age or sex. Therefore, different cutoff numbers of NLN were considered for right-side and left-side colon cancer in our analysis.

Our study has several limitations because of retrospective observation design. First, the coding error and case ascertainment might be possible even we tried to do it best. The retrospective studies always caused bias during data collection and enrollment. The second, although a bigger sample size and adequate follow-up time could be reached during a longer period of data collection, the interference of changes in the treatment strategy and staging system were also present during follow-up. The detailed effects of these changes could not be analyzed in the present study. Third, although we explained our result regarding host immune response in patients with high or low NLN, we did not have microscopic data to support our findings—for example, lymphocyte infiltration and densities of CD3+ and cytotoxic CD8+ T cells. This was a major limitation for our study. The fourth, as discussed above, the two optimal cutoff values of NLN for proximal and distal colon, namely 27 and 12, were determined by plotting the time-dependent ROC curve. The standard cutoff value of NLN was still difficult to establish through such studies. More studies for this issue will be needed in the future.

In addition to the aforementioned limitations, likely unmeasured confounding factors are present in this study. We did not have data regarding tumor molecular subtypes in the present study, including microsatellite instability-high (MSI/dMMR) tumors, RAS, and B-Raf (BRAF) gene mutations. MSI/dMMR occurs due to germline or somatic mutation, which was found in 15–25% of patients [28]. For colorectal cancer, compared with microsatellite stable status, the presence of MSI/dMMR equates to a better prognosis. The mechanism for this is likely associated with immune cell infiltration into the tumor microenvironment [29]. Although MSI/dMMR is a positive prognostic factor for stage II colon cancer after curative resection, the prognostic impact of MSI/dMMR status remains controversial in patients with stage III colon cancer who have received postoperative adjuvant chemotherapy [30–32]. In some conditions, for example, proximal colon cancer or adjuvant chemotherapy with multiple regimens, patients with stage III colon cancer and MSI/dMMR tumors who received oxaliplatin-based adjuvant chemotherapy had better survival outcomes [33, 34]. Moreover, the presence of BRAF status, which occurs in approximately 10% of colorectal cancer, has been considered a predictive marker in the survival of patients with metastatic colorectal cancer because the mutation status correlates with lower therapeutic response to chemotherapy with or without target agents [35]. To our knowledge, the findings concerning the prognostic impact of BRAF mutation in nonmetastatic colon cancer are inconsistent. In a pooled analysis for patients with stage III colon cancer after curative surgery, Taieb et al. noted that BRAF mutation was associated with a relatively short time to relapse, relatively short survival after relapse, and short OS in patients with microsatellite stable status. However, this condition was not noted in patients with MSI/dMMR tumors [36]. On the basis of our data, we could not determine the effect of mixing a good prognostic factor (MSI/dMMR status) and a bad one (BRAF mutation).

## Conclusions

Our data suggested that the high NLN was a positive prognostic factor for stage III colon either in different tumor locations or in subgroups of stage III disease. In advanced stage III colon cancer, the importance of high NLN and their role in anticancer immune response could be highlighted.

## Data Availability

The data included in this study was never presented either as an oral or post presentation before. The raw data supporting our findings cannot be shared because the use of raw data was limited from previous IRB permit.

## Abbreviations

CRC: Colorectal cancer
TNM: The tumor, node, and metastasis stage
NLN: Negative lymph node
SEER: the Surveillance, Epidemiology, and End Result program
CEA: Carcinoembryonic antigen
OS: Overall survival
RFS: Relapse-free survival
ROC: the time-dependent receiver operating characteristic
ELN: Examined lymph node
PLN: Positive lymph node
NLR: Neutrophil-to-lymphocyte ratio
MSI/dMMR: Microsatellite instability-high
RAS: from rat sarcoma virus, a family of related proteins that are expressed in all animal cell lineages and organs
BRAF: B-Raf
